# The Administration of Drugs to Nursing Mothers

**Published:** 1912-09-21

**Authors:** 


### MATERNITY AND MOTHERING.

(Inquiries and Correspondence are invited.)

### The Administration of Drugs to Nursing Mothers.

There are two principal reasons for the administration of medicinal drugs to nursing mothers; first, in order to remedy some abnormal condition of the mother; and, secondly, in order to influence the condition of the child through its food supply. That drugs can be and are passed to some degree out of the system by means of the mother's milk is undoubted; but in the nature of things such a method is speculative, since there is no means (short of expensive analysis) of judging what dose of any drug the suckling will receive. And there are wide divergences of opinion concerning the behaviour of individual drugs in this connection, as well as of the practical utility of this kind of infant therapeutics.

It is, however, fairly certain that some drugs are much more likely to be excreted from the system in the milk than are others. Mothers, therefore, should not be given these drugs for their own disorders or illnesses, unless it is thought desirable that their infants should absorb some of the same substance. Sometimes such a double effect is desirable; as, for instance, in the treatment of constipation, which may affect both the mother and child at the same time. Infants, on the other hand, can be successfully treated by giving these medicines to the mothers, when owing to nasty taste there might be difficulty in getting them to swallow the medicines for themselves.

The following drugs are excreted in the milk, according to a well-known authority on pharmacology:* Oil of anise, oil of dill, oil of turpentine, oil of copaiba, all volatile oils, sulphur, rhubarb, senna, jalap, scammony, castor oil, opium, iodine, indigo, antimony, arsenic, bismuth, iron, lead, mercury, zinc, potassium iodide. Several of these would hardly ever be likely to be required for either a mother or a child; but some of the others are drugs in very frequent use. It will be noticed that several strong aperients are included in the list. Scammony and jalap would seldom be given to a nursing mother; but sulphur, rhubarb, senna, and castor oil are all medicines in common vogue—and, moreover, they are frequently contained in the advertised patent medicines which have such an enormous sale in this country. It must be remembered that constipation is extremely common after childbirth, and that it often requires a somewhat strong purge to overcome it. Castor oil is often advised for this, but it has two disadvantages; for it is not only excreted in the milk, but it also tends to leave behind it a sluggish costiveness, thus perpetuating the very disorder which it at first relieves. It will be noticed that the popular cascara is not included in Dr. Hale White's list: nor is phenol-phthalein, nor tamar indien. The saline aperients also are not included, as is natural when one reflects that their action is mainly localised to the intestine, not due to absorption. With these agents it is seldom that any need for stronger purges arises, especially if enemata are judiciously made use of.

The other principal class of medicament, administered to the mother with the object of influencing the child, is that of anti-syphilitic remedies. It will be observed that in the list given above both mercury and iodides are included. Hence the natural inference that in a case of congenital syphilis both patients, mother and offspring, can be advantageously treated by offering anti-syphilitic remedies to the former alone. Clinically, this hypothesis has been acted on for years by British physicians; but of late it has been called in question a good deal in France, Germany, and the United States.

Several authorities now deny altogether that mercury administered to the mother is excreted in her milk. Somma, of Naples, for instance, after administering mercury to six women by inunction and hypodermically, analysed their milk for prolonged periods and could find no traces of the drug at all. Several authors agree with Somma; and others again contend that the elimination, though it does occur, is so irregular and inconstant that no therapeutic application should be based upon it. Others, again, and a majority, hold that mercury is thus excreted, and can be usefully employed in the treatment of congenital syphilis. To test these conflicting theories Haas has for some years been carrying out observations and experiments which he has now published.†

At first he was chiefly engaged in deciding whether or not syphilitic infants can be benefited by administering mercury to the mother. As a matter of fact he came definitely to the conclusion that they can, although the process is slow; the mother in such cases seems to react to treatment more certainly and more quickly than the child. During these observations he was impressed with the way in which digestive disturbances in the infants disappeared as soon as the mother started to take mercury; so he then tested this effect in other children, not syphilitic, whose digestions were unsatisfactory. He found that vomiting, constipation, diarrhœa, colic, and similar troubles to which infantile flesh is prone, are often speedily relieved by giving the mothers perchloride of mercury in doses of $\frac{1}{32}$ grain three times a day after meals. Two hundred cases were treated; no bad results were encountered in the whole series, and about 35 to 40 per cent. were improved so rapidly and completely that there could be no doubt as to the relation of cause and effect. Even when it fails to benefit, says Dr. Haas, it has thus far proved harmless, and should therefore be given a trial in all cases of gastro-intestinal disturbance in nurslings. It is not a specific, but it is one of the few drugs definitely capable of influencing the metabolism of the mammary glands.

* Dr. W. Hale White: *Materia Medica, Pharmacy, Pharmacology, and Therapeutics.*

† *Archives of Pediatrics*, July 1912.

